# *Ex vivo* DNA Assembly

**DOI:** 10.3389/fbioe.2013.00012

**Published:** 2013-10-21

**Authors:** Adam B. Fisher, Zachary B. Canfield, Laura C. Hayward, Stephen S. Fong, George H. McArthur

**Affiliations:** ^1^Integrative Life Sciences Program, Virginia Commonwealth University, Richmond, VA, USA; ^2^Department of Chemical and Life Science Engineering, Virginia Commonwealth University, Richmond, VA, USA

**Keywords:** DNA assembly, *ex vivo*, end joining, cellular lysates, colorimetric screen, synthetic biology, genetic engineering

## Abstract

Even with decreasing DNA synthesis costs there remains a need for inexpensive, rapid, and reliable methods for assembling synthetic DNA into larger constructs or combinatorial libraries. Advances in cloning techniques have resulted in powerful *in vitro* and *in vivo* assembly of DNA. However, monetary and time costs have limited these approaches. Here, we report an *ex vivo* DNA assembly method that uses cellular lysates derived from a commonly used laboratory strain of *Escherichia coli* for joining double-stranded DNA with short end homologies embedded within inexpensive primers. This method concurrently shortens the time and decreases costs associated with current DNA assembly methods.

## Introduction

Our capacity to (re)engineer living systems is linked to our ability to physically build specific DNA molecules that encode desired functionality and behavior. Traditionally, recombinant DNA has been constructed using restriction cloning (i.e., cutting with an endonuclease and joining with a ligase). In addition to restriction-ligation approaches, site-specific recombination systems have been employed to assemble DNA with great success (Hartley et al., 2000). However, genetic engineering has recently become more flexible with the use of sequence-independent approaches that take advantage of the decreasing cost of DNA synthesis (Li and Elledge, 2007; Gibson et al., 2009; Quan and Tian, 2009, 2011; Zhang et al., 2012). For example, a completely synthetic *Mycoplasma* genome was assembled from chemically synthesized double-stranded DNA (dsDNA) fragments (5–7 kbp each) using an *in vitro* chew-back assembly method in 2008 (Gibson et al., 2008). The authors report that the assembled half-genome DNA molecules could not be propagated by *E. coli* due to their size and therefore relied on yeast transformation-associated recombination (TAR), an *in vivo* assembly in *Saccharomyces cerevisiae*, to finish constructing the genome (Ma et al., 2002). More recently, the mouse mitochondrial genome was reconstructed from overlapping synthetic single-stranded oligonucleotides (60 nucleotides each) using a three-enzyme *in vitro* isothermal DNA assembly method (ISO assembly, also known as Gibson assembly) (Smith et al., 2010).

These homology-based approaches are extremely versatile, but there are disadvantages associated with each. Although isothermal DNA assembly provides a quick and reliable way to simultaneously join together multiple pieces of DNA, the cost of the purified enzymes needed to carry out the assembly reaction is non-trivial (∼US$3/reaction formulated in-house). TAR cloning in *S. cerevisiae* is considerably cheaper but requires: (1) preparation of yeast spheroplasts, (2) the inclusion of both a selection marker and replication origin that function in yeast, and (3) the subsequent purification of the assembled DNA product (if the DNA is intended to be used in an organism other than yeast). This is a long process that takes 8–9 days to complete (Gibson et al., 2008). To address the monetary cost of ISO assembly and the time cost of TAR cloning, we hypothesized that the DNA repair machinery endogenous to yeast and other microorganisms would remain functional within cellular lysates and should be able to catalyze the assembly of linear and circular DNA constructs in just hours.

We tested this hypothesis using cellular lysates derived from *S. cerevisiae, E. coli*, and *Deinococcus radiodurans*, an extremophilic bacterium known to have exceedingly efficient dsDNA repair capabilities. The overall goal of this study was to determine if this general lysate-based approach, termed *ex vivo* DNA assembly, is feasible for physically joining DNA molecules quickly, cheaply, and efficiently. The *ex vivo* assembly reactions were characterized by gel electrophoresis analysis and by a colorimetric screen of the resulting *E. coli* transformants (in which colonies housing the correctly assembled plasmid express a vibrant blue chromoprotein). Here we demonstrate that *ex vivo* dsDNA assembly is organism-dependent (*E. coli* lysate is able to perform end joining, but lysate derived from *S. cerevisiae* or *D. radiodurans* is not) and we show that *in vivo* circularization of overlapping dsDNA occurs after transformation in *E. coli*, a process that is often overlooked when characterizing *in vitro* DNA assembly methods.

## Results

**Figure 1:**
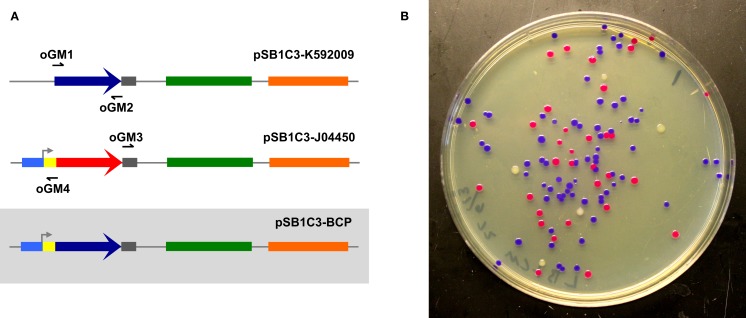
**Two-way *ex vivo* circular DNA assembly**. Two-way assembly was demonstrated by joining a coding sequence for blue chromogenic protein (a 669 bp segment of pSB1C3-K592009 (colored in dark blue) amplified using primers oGM1 and oGM2) and the majority of pSB1C3-J04450 (a 2419 bp segment amplified by primers oGM3 and oGM4), thereby replacing RFP with BCP in pSB1C3-BCP **(A)**. The pMB1 replication origin is colored in green and the chloramphenicol resistance gene is colored orange. Correctly assembled plasmids allow transformants to express BCP (blue colonies) while colonies containing carryover template plasmids appear either red or white **(B)**.

To assess the ability of select cellular lysates to join together dsDNA, we first designed two amplicons with appropriate overlapping ends (26 and 30 bp overlaps) to be assembled into a circular plasmid (Figure 1A). Correctly assembled plasmids endow *E. coli* transformants with selective resistance to the antibiotic chloramphenicol and also visually screenable expression of a blue chromogenic protein (BCP) native to the coral *Acropora millepora* (Figure 1B) (Alieva et al., 2008). Template plasmids housing the *bcp* coding sequence (pSB1C3-K592009) and the appropriate antibiotic resistance and replication origin (pSB1C3-J04450) produce white and red/pink colonies, respectively, providing a convenient way to track transformation efficiencies (Figure 1B). In this initial experiment, we used lysates of *S. cerevisiae, E. coli*, and *D. radiodurans* (hereafter *Sce, Eco*, and *Dra*, respectively) and a simple buffer containing ATP and MgCl_2_ to attempt *ex vivo* DNA assembly. The two amplicons were incubated for 1 h with each lysate. Samples of each reaction mixture were subsequently used to transform *E. coli* NEB10β.

Interestingly, *Dra*-incubated DNA did not produce transformants, although DNA incubated with *Sce* or *Eco* successfully transformed *E. coli*. Further analysis by agarose gel electrophoresis showed significant degradation of the individual amplicons, indicating that a highly active exonuclease system might have prevented the assembly of DNA by *Dra* (Figure S1 in Supplementary Material). Even expected background transformants resulting from lingering circular PCR template were absent, suggesting that endonuclease activity is also high in *Dra*. Indeed, after further investigation we found a consensus *Drd*I site (GAC-N_6_-GTC) between 1475 and 1486 (GACGCTCAAGTC) in the replication origin of our plasmids (Polisson and Morgan, 1989).

**Figure 2:**
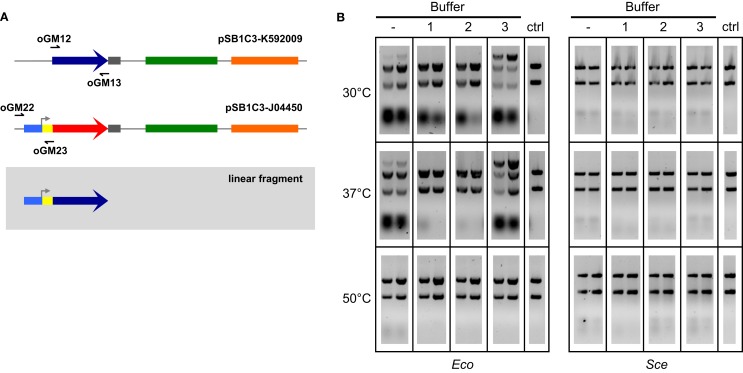
**Two-way *ex vivo* linear DNA assembly**. Optimized *ex vivo* reaction conditions were identified by visualizing the joining of a 697 bp segment of pSB1C3-K592009 (amplified by oGM12 and oGM13) and a 381 bp segment of pSB1C3-J04450 (amplified by oGM22 and oGM23) into a larger linear DNA molecule **(A)**. Reactions were run for 1 and 2 h (left and right in each gel, respectively), at three temperatures and four buffer compositions for each *Eco* and *Sce* **(B)**. All buffers contained 1 mM NAD and 1 mM DTT. ATP and Mg^2+^ concentrations (mM:mM) varied as follows: (1) 10:5, (2) 5:5, (3) 1:10. The control lanes include a reaction with no supplemented buffer (−) and a negative control (“ctrl”) DNA-only lane (no buffer or lysate).

We then explored ways to improve the efficiency of *ex vivo* DNA assembly with *Eco* and *Sce* by varying the relative composition of ATP and MgCl_2_ in our buffer, adding NAD to the buffer to power NAD^+^-dependent processes such as ligation, modulating the temperature at which the reaction mixture was incubated, increasing the amount of cellular lysate in our reaction, and increasing the duration of incubation. To directly assess the efficacy of *ex vivo* DNA assembly and avoid variation associated with transformation, we chose to visualize formation of a linear product (Figure 2A) from a pair of overlapping amplicons (28 bp overlap) via agarose gel electrophoresis of the *ex vivo* assembly reaction prior to transformation (Figure 2B). In this manner, we were able to compare different reaction conditions on their ability to join two overlapping dsDNA into one linear product.

Buffers were made of 1 mM DTT, 1 mM NAD, and varying concentrations of ATP and Mg^2+^. A wide range of ATP:Mg^2+^ ratios were initially tested with 1-h *Eco* reactions at 37°C: 1:5, 1:10, 1:20, 5:5, 5:10, 5:20, 10:5, 10:10, and 10:20 (mM:mM). Only three ATP:Mg^2+^ ratios were chosen for further reaction optimization: 10:5, 5:5, and 1:10 (mM:mM), buffers 1–3 in Figure 2B, respectively). Linear products resulting from assembly by *Sce* were not at all visible in the gel, although bands of small pieces of DNA indicate that there is some nuclease activity under most conditions tested. On the other hand, *Eco* appears to have significant activity at 30 and 37°C even without the addition of buffer. Previous studies have noted that the nucleolytic behavior of the RecBCD complex of *E. coli* changes based upon the relative amount of ATP to free Mg^2+^ *in vitro* (Taylor and Smith, 1995). We observed a similar trend in our lysates; the most efficient assembly reactions (for both 30 and 37°C) were carried out under conditions of excess magnesium relative to ATP (Buffer 3 in Figure 2B), probably because ATP can chelate Mg^2+^ with high affinity under physiological conditions (Wilson and Chin, 1991).

Each *ex vivo* reaction condition was tested for both 1- and 2-h incubations. Initial experiments indicated that greater incubation times (3–6 h) do not improve *ex vivo* assembly yields (data not shown). For all successful assemblies, 2-h reactions appear to generate more linear product than 1-h reactions. The temperatures selected for *ex vivo* reaction optimization reflect cell culture conditions (30°C for yeast and 37°C for *E. coli*) and the temperature used for ISO assembly reactions (50°C). The 50°C reactions are considered a negative control since we do not expect the cellular machinery in *Eco* or *Sce* to be thermostable, although transient activity may occur initially. Based on the results of these experiments we selected a buffer composition of 1 mM ATP, 10 mM Mg^2+^, 1 mM DTT, and 1 mM NAD^+^ (i.e., Buffer 3 in Figure 2B) incubated at 37°C for 2 h as our optimized conditions for both *Eco* and *Sce*.

**Figure 3:**
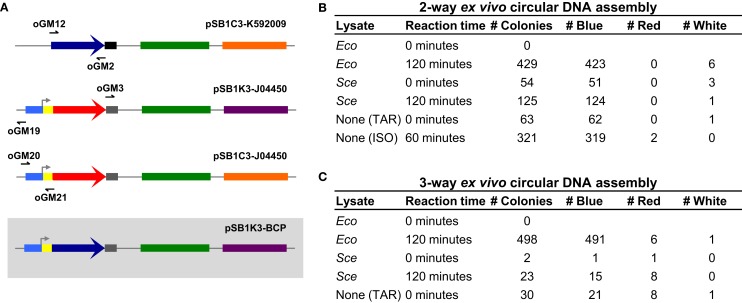
**Three-way *ex vivo* circular DNA assembly and colony counts for two- and three-way assembly**. Three-way assembly was demonstrated by joining the BCP coding sequence (a 696 bp segment of pSB1C3-K592009 amplified by oGM12 and oGM2), the majority of a plasmid carrying a different antibiotic resistance gene (a 2214 bp segment of pSB1K3-J04450 amplified by oGM3 and oGM19) and a promoter-RBS region used to drive BCP expression (a 387 bp segment of pSB1C3-J04450 amplified by oGM20 and oGM21), resulting in pSB1K3-BCP **(A)**. The pMB1 replication origin is colored in green, the chloramphenicol resistance gene is colored orange and the kanamycin resistance gene is colored purple. Colony counts for optimized two- and three-way assembly experiments **(B,C)**. The negative control assembly reaction is labeled “None (TAR)” meaning that these colonies result from *in vivo* assembly in *E. coli*. The two-way positive control is an isothermal assembly reaction and is labeled “None (ISO).”

Under optimized reaction conditions, *Eco* and *Sce* were used again to perform the two-way dsDNA assembly. In addition, we designed three overlapping amplicons (30, 29, and 26 bp overlaps) to demonstrate a three-way assembly, which is not only more complex but also more useful for generating combinatorial libraries (Figure 3A). For each of these assembly tests, lysate-incubated DNA was allowed to react for 2 h before transformation of *E. coli*. Control conditions of zero incubation time and reactions with no lysate added were also run to ensure that the lysate was indeed facilitating DNA assembly and not otherwise affecting the transformation process.

Our results (Figures 3B and 3C) clearly indicate that DNA is indeed being assembled *ex vivo*. For two- and three-way assemblies, both *Eco* and *Sce*, transformation efficiencies significantly increase when the DNA to be assembled is allowed to incubate with the lysate. Unexpectedly, the “no lysate” negative control revealed that for two- and three-way assemblies the overlapping amplicons can be joined together into a circular plasmid *in vivo*, suggesting that a significant fraction of the *ex vivo* assembly reactions – and also *in vitro* reactions (e.g., ISO assembly) – actually occur inside *E. coli* after transformation (i.e., TAR cloning in *E. coli*). Although *Eco*-mediated assembly produced many blue colonies for both two- and three-way assemblies (423 and 491 colonies, respectively), *Eco* appeared to be detrimental to the overall transformation efficiency when it was not allowed to incubate with the DNA amplicons.

To test whether or not the detergent-based lysis buffer used to produce *Eco* was affecting the transformation process, we carried out the assembly reactions and transformations in the presence of the lysis buffer but without cellular lysate. As summarized in Table S1 in Supplementary Material, the bacterial lysis buffer inhibits transformation completely. Therefore, *Eco*-mediated assembly is likely much more efficient than we have observed and an alternative lysis method would probably increase overall transformation efficiency. The yeast lysis buffer does not appear to inhibit transformation of *E. coli*, which is not surprising because it is designed to lyse yeast cells. Interestingly, the number of colonies produced by DNA incubated with yeast lysis buffer was statistically identical to the number of colonies produced by *Sce*-incubated DNA and that produced by the “no lysate” control. These data suggests that *Sce*-mediated DNA assembly was not observed but rather indicates that *in vivo* end joining in *E. coli* facilitates DNA assembly in this case.

## Discussion

The assembly of DNA sequences in novel combinations is fundamental to genetic engineering and molecular biology research. We sought to develop an inexpensive and efficient method for DNA assembly by using cellular lysates instead of purified enzymes to perform DNA end joining. Through a series of experiments investigating circular and linear DNA assembly, we found that: (1) lysate derived from *E. coli* NEB10β, a RecA-deficient strain, was able to efficiently assemble dsDNA, (2) a fraction of the assembly takes places inside *E. coli* NEB10β post-transformation by *in vivo* DNA assembly, and (3) lysates derived from *D. radiodurans* and *S. cerevisiae* were not able to join together dsDNA under the conditions we tested. In addition, it is worth highlighting that we attempted single-stranded DNA (ssDNA) assembly of synthetic 60-mer oligonucleotides (20 bp overlaps) that was accomplished by ISO assembly but was unsuccessful using our *ex vivo* approach (data not shown). Over the course of all of our experiments we found that the trends in efficiency of assembly held true across organisms independent of batch-to-batch variation.

To assess the efficacy of *ex vivo* DNA assembly, we used a combination of analytical gel electrophoresis for visualizing linear product and a novel colorimetric screen for easily identifying colonies containing assembled circular DNA. The colorimetric screen is particularly useful because it allows the simultaneous detection of desired DNA assembly (correctly assembled plasmids express BCP, making *E. coli* colonies appear blue/purple) and determination of transformation efficiency between trials by tracking carryover template plasmids (pSB1C3-K592009 produces typical white colonies while pSB1C3-J04450 and pSB1K3-J04450 produce red colonies by expressing mRFP1). In this case, template plasmid digestion by *Dpn*I is not preferred. We anticipate that the design of similar colorimetric screens, in which individual template plasmids can either be selected against or uniquely identified by color, will assist in the development of superior DNA assembly methods and aid in the study of natural DNA repair mechanisms.

In *E. coli*, double-strand break (DSB) repair, dominated by homologous recombination, relies on the RecBCD complex to expose ssDNA ends and load RecA, forming the nucleofilament that subsequently directs a homology search and strand invasion of dsDNA (Chen et al., 2008). Previously, it was shown that *recA*^−^ strains of *E. coli* join linear dsDNA with homologous overhangs in lysate more efficiently than their RecA-expressing counterparts. As *recBCD*^−^ strains have also been reported to direct accurate DNA end joining, *ex vivo* assembly reactions may involve mechanisms independent of homologous recombination machinery, perhaps by an alternative end-joining pathway (Chayot et al., 2010). We suspect that both *ex vivo* and *in vivo* DNA assembly is likely facilitated by multiple competing DNA repair mechanisms, but largely by RecBCD, RecJ, and RecQ.

The radio-resistant bacterium *D. radiodurans* has been shown to completely reassemble its genome from hundreds of radiation-induced DSBs in a matter of 3–4 h, using multiple copies of its genome as templates to facilitate accurate repair by homologous recombination (Cox and Battista, 2005). Intriguingly, *D. radiodurans* lacks any homolog to RecB or RecC, seemingly relying on the helicase, exonuclease, and RecA-loading activity of RecQ, RecJ, and the RecFOR system, respectively – although a RecA-independent system is known to exist as well (Daly and Minton, 1996; Jiao et al., 2012). Disappointingly, *Dra* consistently failed to yield transformants from *ex vivo* assembly reactions. Analysis of the reaction mixture by gel electrophoresis revealed high exonuclease activity indicated by the complete disappearance of the amplicon bands by 2 h (Figure S1 in Supplementary Material). More puzzling was the absence of background transformations by carryover plasmid DNA used as template in the PCR step, which indicated endonucleolytic activity by known and possibly unknown nucleases. While this constitutes a design consideration going forward, we hypothesize that the excessive exonuclease activity must be attenuated for *D. radiodurans* lysate to serve as an efficient catalyst for *ex vivo* DNA assembly.

The robust homologous recombination system and additional end-joining pathways found in *S. cerevisiae* make it a powerful host for *in vivo* DNA assembly. Indeed, TAR cloning in yeast has resulted in a number of successful large-scale feats of genetic engineering including construction of ∼454 kbp of the high G + C content genome of *Synechococcus elongatus* (Noskov et al., 2012). Therefore, it was surprising that *Eco* outperformed *Sce* in DNA end-joining reactions. It is possible that the buffer conditions that work well for *Eco* may not be optimal for the protein complexes in *Sce*, although we suspect there is a higher concentration of DNA repair proteins in *Eco* than *Sce* based on culture and lysis conditions. Therefore, DNA assembly with *Sce* may be limited by mass transport (not protein activity), which can be improved upon by synchronizing the cell cycle of the culture and/or harvesting more yeast cells.

While all organisms possess some ability to repair DSBs (Blackwood et al., 2013), we made several observations in the selection of organisms for *ex vivo* DNA assembly. Lysate derived from *D. radiodurans* was an exciting prospect for DNA assembly but clearly suffered from excessive nuclease activity. The cloning problems associated with endonuclease activity cannot be avoided without sequence design constraints (for sequence-specific endonucleases) or by generating a nuclease knockout strain, which may be exceedingly difficult in a genetically intractable organism such as *D. radiodurans*. Exonuclease activity is required for DNA end resection, but is a balancing act. We saw in *Sce* very little exonuclease activity, which preserves the regions of homology, but may not expose complementary overhangs. Conversely, extreme exonuclease activities, like that of *Dra*, may be resecting bi-directionally, deleting the necessary homologous regions. Again, knockouts of single-strand exonucleases implicated in deletion of exposed overhangs may greatly improve the efficiency of end joining (Dutra et al., 2007). Another strategy to improve efficiency may be to increase the lengths of homology on the fragments. Previous *in vivo* studies on single-strand annealing in *S. cerevisiae* showed an approximately linear dependence on homology up to 415 bp with sequence homology as short as 29 bp used 0.2% of the time (Sugawara et al., 2000). In our work, we chose to use small overlapping regions because these can be embedded within short, inexpensive primers (<60 mer). Haploid hosts, like *E. coli*, do not generally possess highly active end-joining mechanisms. Instead they rely on *recA*-dependent recombination to direct exposed ssDNA nucleofilaments toward homologous dsDNA. By using *recA*^−^ strains we postulate that our resected single-stranded ends are now permitted to anneal with other exposed complementary single-stranded ends rather than dsDNA templates. Directing recombination toward this single-strand annealing pathway may prove even more successful with microbes which more highly express these alternate end-joining mechanisms (Gupta et al., 2013). Using an *ex vivo* approach, the diversity of culturable microorganisms can be mined and quickly assayed for their ability to perform DNA end joining, exposing deeper insight into the mechanisms of DNA repair and new platforms for cloning.

The idea that genetic engineers can use native DNA repair mechanisms to build recombinant DNA molecules is not new. However, access to relatively cheap synthetic dsDNA has only recently become a reality. As DNA synthesis becomes less expensive, the majority of time and cost considerations will be spent on DNA assembly steps. Continued development of faster and cheaper DNA assembly methods, such as the *ex vivo* DNA assembly described here, will further accessibility to DNA-based studies and applications. Of course, there are limitations to *ex vivo* DNA assembly as with any approach. For example, all homology-based assembly methods are constrained by the fact that there must be overlapping homologous ends. While the overlapping lengths utilized here are easily designed and embedded in primers, it is impossible to generate genetic variants in which the diversity occurs within the overlapping region. In addition, ssDNA assembly appears to be better achieved by ISO assembly or polymerase chain assembly (PCA). Outside of these small design constraints, *ex vivo* DNA assembly should be a very useful method for genetic engineers and will hopefully contribute to lowering the monetary and time costs associated with building DNA.

## Materials and Methods

### Generation of DNA fragments for *ex vivo* DNA assembly

The DNA fragments used to demonstrate *ex vivo* DNA assembly were generated using standard PCR of parts of the following plasmids from the BioBricks registry: pSB1C3-J04450, pSB1C3-K592009, and pSB1K3-J04450. Primers used in this study (Table S2 in Supplementary Material) were generated using the j5 automated DNA assembly software (Chen et al., 2012; Ham et al., 2012; Hillson et al., 2012). Amplicons were generated by 100 μL PCR reactions with Q5 polymerase (NEB) under standard reaction conditions. These reactions were cycled at 98°C for 30 s; 98°C for 10 s, 50°C for 15 s, 72°C for 25 s (repeated for 25 cycles); 72°C for 2:00. To purify these PCR reactions, samples were run on 1.0% agarose-TAE gels stained with GelGreen (Biotium™). The gels were run at 100 V for 30 min and subsequently visualized under blue light. The gels showed no side product formation and bands were excised and isolated using ZymoClean™ Gel DNA Recovery Kit (Zymo Research).

### Preparation of cellular lysates

Cellular lysates were prepared from the following strains: *E. coli* NEB10β (NEB #C3019), *S. cerevisiae* BY4741 and *D. radiodurans* R1 (ATCC^®^ 13939). *E. coli* was grown in Terrific Broth with glycerol (Sigma) at 37°C with shaking at 250 rpm. *D. radiodurans* was grown in 123 TGY medium (5% Tryptone, 5% Yeast extract, 1% Glucose, 1% Potassium monophosphate) at 30°C and *S. cerevisiae* was grown with YPD media (Sigma) at 30°C, both shaking at 250 rpm. The preparation of the bacterial (*E. coli* and *D. radiodurans*) and the *S. cerevisiae* lysates varied slightly: bacterial cultures were pelleted once the OD_600 nm_ reading was between 6.00 and 6.50, while the yeast cultures were pelleted once they reached between 4.00 and 4.50 OD_600 nm_. Volumes of 4–6 mL of culture were centrifuged at 13,200 rpm at 4°C for 2 min, washed with 1 mL of Milli-Q H_2_O, centrifuged again and the wet pellet massed. 2X CelLytic B Lysis Reagent (Sigma) was added to the bacterial cell pellets at 3 μL/mg of cells. 1X CelLytic Y Lysis Reagent (Sigma) was added to the yeast pellets in the same ratio. After the addition of the lysis reagents, the cells were incubated at 37°C for 10 min shaking at 300 rpm. The lysed cells were centrifuged at 13,200 rpm for 15 min and a 20 μL sample of the supernatant (lysate) was mixed with 20 μL of 100% glycerol to yield 40 μL aliquots. All lysate aliquots were stored at −20°C.

### DNA assembly reactions, agarose gel analyses of assembled DNA, and transformations

Buffers for assembly reactions were prepared from 100× stock solutions. Stock solutions were as follows: 100 mM NADβ, 100 mM ATP, 100 mM DTT, 1 M MgCl2 (Symington et al., 1983). Tris-HCl was added to the buffer to 500 mM from a 1 M stock solution. A typical assembly reaction would include 6 μL of cellular lysate, 2 μL of 10× buffer, 2 μL of nuclease free water (Ambion^®^), and 10 μL of a DNA master mix. DNA master mixes contained all fragments needed for assembly and were formulated with 20 ng/μL of the backbone and 6:1 molar ratio of insert fragments to the backbone. Gel analysis was performed using 1.0–1.2% agarose-TAE gels containing GelRed (Biotium) as the staining agent. All analytical gels were run at 100 V for 30–50 min and visualized with a Molecular Imager^®^ Gel Doc™ XR+ Imaging System with Image Lab™ v4.0 software (Bio-Rad). Transformations were performed using chemically competent *E. coli* NEB10β (NEB) according to manufacturer’s recommendations. Briefly, 2 μL of reaction mixture (from *ex vivo* assemblies) or diluted DNA master mix (for *in vivo* assembly) were added to each transformation, these were incubated for 30 min on ice, heat shocked at 42°C, recovered in 950 μL SOC at 37°C for 60 min and 50 μL of culture was spread onto agar plates containing the appropriate antibiotic.

## Author Contributions

George H. McArthur and Adam B. Fisher designed the experiments, which were carried out in the laboratory of Stephen S. Fong. Adam B. Fisher performed the experiments with Zachary B. Canfield and Laura C. Hayward. George H. McArthur, Adam B. Fisher, and Stephen S. Fong interpreted the data and wrote the manuscript. All authors discussed results and commented on the manuscript.

## Conflict of Interest Statement

The authors declare that the research was conducted in the absence of any commercial or financial relationships that could be construed as a potential conflict of interest.

## Supplementary Material

The Supplementary Material for this article can be found online at http://www.frontiersin.org/Journal/10.3389/fbioe.2013.00012/abstract
