# Supplementary material for: Ex vivo DNA Assembly
Source: Front Bioeng Biotechnol. 2013 Oct 21;1:12. doi: 10.3389/fbioe.2013.00012 (PMC4090908; doi:10.3389/fbioe.2013.00012)
Supplement: Supplementary file 1 [file 67930_Fong_DataSheet1.DOCX]

***Supplementary information***

***ex vivo* DNA assembly**

Adam B. Fisher^2^, Zachary B. Canfield^1^, Laura C. Hayward^1^, Stephen S. Fong^1,2,★^ and George H. McArthur IV^1,★^

Department of Chemical and Life Science Engineering^1^ and Integrative Life Sciences Program^2^

Virginia Commonwealth University, Richmond, VA, USA

^★^ Correspondence should be addressed to G.H.M. (mcarthurgh@vcu.edu) or S.S.F. (ssfong@vcu.edu)

Virginia Commonwealth University

Chemical and Life Science Engineering

601 W. Main Street

Richmond, VA, 23284-3028, USA

Figure S1 below shows the initial 2-way *ex vivo* dsDNA assembly reactions using lysates derived from *D. radiodurans*, *S. cerevisiae* and *E. coli* (*Dra*, *Sce* and *Eco*, respectively). Each lysate was tested for three different reaction durations (30, 60 and 120 minutes, from left to right). It is clear from the gel that *Dra* degrades DNA as a function of time; the DNA fragments are nearly gone after being incubated for two hours with *Dra*. DNA incubated with *Sce* or *Eco* produced transformants – although no circularized product can be seen in the gel here.


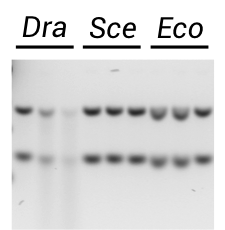


**Figure S1 | Incubation of DNA with *Dra*, *Sce* and *Eco* for initial *ex vivo* DNA assembly attempt**

Supplementary Table 1 is a tabulation of colony counts for transformations that were carried out in the presence of lysis buffer and the appropriate DNA but without cellular lysate. No transformants were seen when the DNA was incubated with the bacterial lysis buffer (CelLytic B), suggesting that this particular buffer somehow inhibits transformation. On the other hand, the yeast lysis buffer (CelLytic Y) showed no inhibition of transformation, which may not be surprising since the bacterial lysis buffer is designed to act on bacteria such as *E. coli* NEB10β. However, it is also clear from these results that *Sce* does not offer any advantage over the yeast lysis buffer for DNA assembly and, therefore, it can be deduced that the transformants resulting from *Sce* incubation are actually a result of *in vivo* DNA assembly in *E. coli*.

**Table S1 | Lysis buffer impacts transformation efficiency**

| **2-way *ex vivo* circular DNA assembly** | | | | | |
| --- | --- | --- | --- | --- | --- |
| **Lysis buffer** | **Reaction time** | **# Colonies** | **# Blue** | **# Red** | **# White** |
| CelLytic B | 0 minutes | 0 |  |  |  |
| CelLytic B | 120 minutes | 0 |  |  |  |
| CelLytic Y | 0 minutes | 42 | 41 | 0 | 1 |
| CelLytic Y | 120 minutes | 150 | 140 | 0 | 10 |
|  |  |  |  |  |  |
|  |  |  |  |  |  |
| **3-way *ex vivo* circular DNA assembly** | | | | | |
| **Lysis buffer** | **Reaction time** | **# Colonies** | **# Blue** | **# Red** | **# White** |
| CelLytic B | 0 minutes | 0 |  |  |  |
| CelLytic B | 120 minutes | 0 |  |  |  |
| CelLytic Y | 0 minutes | 15 | 8 | 7 | 0 |
| CelLytic Y | 120 minutes | 27 | 18 | 9 | 0 |

**Table S2 | Primers used in this study**

| **Primer** | **Sequence** | **Length** |
| --- | --- | --- |
| oGM1 | AGAGAAAGAGGAGAAAATGAGTGTGATCGCTAAACAAATGACCTACAAGG | 50 |
| oGM2 | ATTTGATGCCTGGTTATTAGGCGACCACAGGTTTGCGTGC | 40 |
| oGM3 | GGTCGCCTAATAACCAGGCATCAAATAAAACGAAAGGCT | 39 |
| oGM4 | GCGATCACACTCATTTTCTCCTCTTTCTCTAGTATGTGTGA | 41 |
| oGM12 | AGGAGAAATACTAGATGAGTGTGATCGCTAAACAAATGACCTACAAGG | 48 |
| oGM19 | TGGTTTCTTAGAAGCTGATCCTTCAACTCAGCA | 33 |
| oGM20 | TGGTTTCTTAGAAGCTGATCCTTCAACTCAGCA | 50 |
| oGM21 | AGCGATCACACTCATCTAGTATTTCTCCTCTTTCTCTAGTATGTG | 45 |
| oGM22 | TGAAGGATCAGCTTCTAAGAAACCATTATTATCATGACATTAACC | 45 |
| oGM23 | GCGATCACACTCATCTAGTATTTCTCCTCTTTCTCTAGTATGTG | 44 |

**Plasmid sequences** – please contact GM (mcarthurgh@vcu.edu) for Genbank files. The -10 and -35 hexamer elements of the constitutive promoter P_R0010_ are double underlined. Relevant features are colored coded.

*pSB1C3-K592009* (2739 bp)


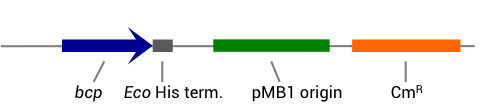


GAATTCGCGGCCGCTTCTAGAGATGAGTGTGATCGCTAAACAAATGACCTACAAGGTTTATATGTCAGGCACGGTCAATGGACACTACTTTGAGGTCGAAGGCGATGGAAAAGGTAAGCCCTACGAGGGGGAGCAGACGGTAAAGCTCACTGTCACCAAGGGCGGACCTCTGCCATTTGCTTGGGATATTTTATCACCACAGTGTCAGTACGGAAGCATACCATTCACCAAGTACCCTGAAGACATCCCTGACTATGTAAAGCAGTCATTCCCGGAGGGCTATACATGGGAGAGGATCATGAACTTTGAAGATGGTGCAGTGTGTACTGTCAGCAATGATTCCAGCATCCAAGGCAACTGTTTCATCTACCATGTCAAGTTCTCTGGTTTGAACTTTCCTCCCAATGGACCTGTCATGCAGAAGAAGACACAGGGCTGGGAACCCAACACTGAGCGTCTCTTTGCACGAGATGGAATGCTGCTAGGAAACAACTTTATGGCTCTGAAGTTAGAAGGAGGCGGTCACTATTTGTGTGAATTTAAAACTACTTACAAGGCAAAGAAGCCTGTGAAGATGCCAGGGTATCACTATGTTGACCGCAAACTGGATGTAACCAATCACAACAAGGATTACACTTCGGTTGAGCAGTGTGAAATTTCCATTGCACGCAAACCTGTGGTCGCCTAATAATACTAGTAGCGGCCGCTGCAGTCCGGCAAAAAA**GGGCAAGGTGTCACCACCCTGCCCTTTTTCTTTAAAACCGAAAAGATTACTTCGCGTTAT**GCAGGCTTCCTCGCTCACTGACTCGCTGCGCTCGGTCGTTCGGCTGCGGCGAGCGGTATCAGCTCACTCAAAGGCGGTAATACGGTTATCCACAGAATCAGGGGATAACGCAGGAAAGAACATGTGAGCAAAAGGCCAGCAAAAGGCCAGGAACCGTAAAAAGGCCG**CGTTGCTGGCGTTTTTCCACAGGCTCCGCCCCCCTGACGAGCATCACAAAAATCGACGCTCAAGTCAGAGGTGGCGAAACCCGACAGGACTATAAAGATACCAGGCGTTTCCCCCTGGAAGCTCCCTCGTGCGCTCTCCTGTTCCGACCCTGCCGCTTACCGGATACCTGTCCGCCTTTCTCCCTTCGGGAAGCGTGGCGCTTTCTCATAGCTCACGCTGTAGGTATCTCAGTTCGGTGTAGGTCGTTCGCTCCAAGCTGGGCTGTGTGCACGAACCCCCCGTTCAGCCCGACCGCTGCGCCTTATCCGGTAACTATCGTCTTGAGTCCAACCCGGTAAGACACGACTTATCGCCACTGGCAGCAGCCACTGGTAACAGGATTAGCAGAGCGAGGTATGTAGGCGGTGCTACAGAGTTCTTGAAGTGGTGGCCTAACTACGGCTACACTAGAAGAACAGTATTTGGTATCTGCGCTCTGCTGAAGCCAGTTACCTTCGGAAAAAGAGTTGGTAGCTCTTGATCCGGCAAACAAACCACCGCTGGTAGCGGTGGTTTTTTTGTTTGCAAGCAGCAGATTACGCGCAGAAAAAAAGGATCTCAAGAAGATCCTTTGA**TCTTTTCTACGGGGTCTGACGCTCAGTGGAACGAAAACTCACGTTAAGGGATTTTGGTCATGAGATTATCAAAAAGGATCTTCACCTAGATCCTTTTAAATTAAAAATGAAGTTTTAAATCAATCTAAAGTATATATGAGTAAACTTGGTCTGACAGCTCGAGGCTTGGATTCTCACCAATAAAAAACGCCCGGCGGCAACCGAGCGTTCTGAACAAATCCAGATGGAGTTCTGAGGTCATTACTGGATCTATCAACAGGAGTCCAAGCGAGCTCGATATCAAA**TTACGCCCCGCCCTGCCACTCATCGCAGTACTGTTGTAATTCATTAAGCATTCTGCCGACATGGAAGCCATCACAAACGGCATGATGAACCTGAATCGCCAGCGGCATCAGCACCTTGTCGCCTTGCGTATAATATTTGCCCATGGTGAAAACGGGGGCGAAGAAGTTGTCCATATTGGCCACGTTTAAATCAAAACTGGTGAAACTCACCCAGGGATTGGCTGAGACGAAAAACATATTCTCAATAAACCCTTTAGGGAAATAGGCCAGGTTTTCACCGTAACACGCCACATCTTGCGAATATATGTGTAGAAACTGCCGGAAATCGTCGTGGTATTCACTCCAGAGCGATGAAAACGTTTCAGTTTGCTCATGGAAAACGGTGTAACAAGGGTGAACACTATCCCATATCACCAGCTCACCGTCTTTCATTGCCATACGAAATTCCGGATGAGCATTCATCAGGCGGGCAAGAATGTGAATAAAGGCCGGATAAAACTTGTGCTTATTTTTCTTTACGGTCTTTAAAAAGGCCGTAATATCCAGCTGAACGGTCTGGTTATAGGTACATTGAGCAACTGACTGAAATGCCTCAAAATGTTCTTTACGATGCCATTGGGATATATCAACGGTGGTATATCCAGTGATTTTTTTCTCCATTTTAGCTTCC**TTAGCTCCTGAAAATCTCGATAACTCAAAAAATACGCCCGGTAGTGATCTTATTTCATTATGGTGAAAGTTGGAACCTCTTACGTGCCCGATCAACTCGAGTGCCACCTGACGTCTAAGAAACCATTATTATCATGACATTAACCTATAAAAATAGGCGTATCACGAGGCAGAATTTCAGATAAAAAAAATCCTTAGCTTTCGCTAAGGATGATTTCTG

*pSB1C3-J04450* (3139 bp)


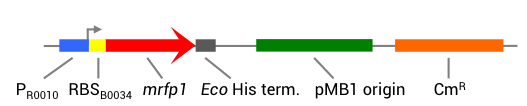


GAATTCGCGGCCGCTTCTAGAG**CAATACGCAAACCGCCTCTCCCCGCGCGTTGGCCGATTCATTAATGCAGCTGGCACGACAGGTTTCCCGACTGGAAAGCGGGCAGTGAGCGCAACGCAATTAATGTGAGTTAGCTCACTCATTAGGCACCCCAGGCTTTACACTTTATGCTTCCGGCTCGTATGTTGTGTGGAATTGTGAGCGGATAACAATTTCACACA**TACTAGAGAAAGAGGAGAAATACTAGATGGCTTCCTCCGAAGACGTTATCAAAGAGTTCATGCGTTTCAAAGTTCGTATGGAAGGTTCCGTTAACGGTCACGAGTTCGAAATCGAAGGTGAAGGTGAAGGTCGTCCGTACGAAGGTACCCAGACCGCTAAACTGAAAGTTACCAAAGGTGGTCCGCTGCCGTTCGCTTGGGACATCCTGTCCCCGCAGTTCCAGTACGGTTCCAAAGCTTACGTTAAACACCCGGCTGACATCCCGGACTACCTGAAACTGTCCTTCCCGGAAGGTTTCAAATGGGAACGTGTTATGAACTTCGAAGACGGTGGTGTTGTTACCGTTACCCAGGACTCCTCCCTGCAAGACGGTGAGTTCATCTACAAAGTTAAACTGCGTGGTACCAACTTCCCGTCCGACGGTCCGGTTATGCAGAAAAAAACCATGGGTTGGGAAGCTTCCACCGAACGTATGTACCCGGAAGACGGTGCTCTGAAAGGTGAAATCAAAATGCGTCTGAAACTGAAAGACGGTGGTCACTACGACGCTGAAGTTAAAACCACCTACATGGCTAAAAAACCGGTTCAGCTGCCGGGTGCTTACAAAACCGACATCAAACTGGACATCACCTCCCACAACGAAGACTACACCATCGTTGAACAGTACGAACGTGCTGAAGGTCGTCACTCCACCGGTGCTTAATAACGCTGATAGTGCTAGTGTAGATCGCTACTAGAGCCAGGCATCAAATAAAACGAAAGGCTCAGTCGAAAGACTGGGCCTTTCGTTTTATCTGTTGTTTGTCGGTGAACGCTCTCTACTAGAGTCACACTGGCTCACCTTCGGGTGGGCCTTTCTGCGTTTATATACTAGTAGCGGCCGCTGCAGTCCGGCAAAAAA**GGGCAAGGTGTCACCACCCTGCCCTTTTTCTTTAAAACCGAAAAGATTACTTCGCGTTAT**GCAGGCTTCCTCGCTCACTGACTCGCTGCGCTCGGTCGTTCGGCTGCGGCGAGCGGTATCAGCTCACTCAAAGGCGGTAATACGGTTATCCACAGAATCAGGGGATAACGCAGGAAAGAACATGTGAGCAAAAGGCCAGCAAAAGGCCAGGAACCGTAAAAAGGCCG**CGTTGCTGGCGTTTTTCCACAGGCTCCGCCCCCCTGACGAGCATCACAAAAATCGACGCTCAAGTCAGAGGTGGCGAAACCCGACAGGACTATAAAGATACCAGGCGTTTCCCCCTGGAAGCTCCCTCGTGCGCTCTCCTGTTCCGACCCTGCCGCTTACCGGATACCTGTCCGCCTTTCTCCCTTCGGGAAGCGTGGCGCTTTCTCATAGCTCACGCTGTAGGTATCTCAGTTCGGTGTAGGTCGTTCGCTCCAAGCTGGGCTGTGTGCACGAACCCCCCGTTCAGCCCGACCGCTGCGCCTTATCCGGTAACTATCGTCTTGAGTCCAACCCGGTAAGACACGACTTATCGCCACTGGCAGCAGCCACTGGTAACAGGATTAGCAGAGCGAGGTATGTAGGCGGTGCTACAGAGTTCTTGAAGTGGTGGCCTAACTACGGCTACACTAGAAGAACAGTATTTGGTATCTGCGCTCTGCTGAAGCCAGTTACCTTCGGAAAAAGAGTTGGTAGCTCTTGATCCGGCAAACAAACCACCGCTGGTAGCGGTGGTTTTTTTGTTTGCAAGCAGCAGATTACGCGCAGAAAAAAAGGATCTCAAGAAGATCCTTTGA**TCTTTTCTACGGGGTCTGACGCTCAGTGGAACGAAAACTCACGTTAAGGGATTTTGGTCATGAGATTATCAAAAAGGATCTTCACCTAGATCCTTTTAAATTAAAAATGAAGTTTTAAATCAATCTAAAGTATATATGAGTAAACTTGGTCTGACAGCTCGAGGCTTGGATTCTCACCAATAAAAAACGCCCGGCGGCAACCGAGCGTTCTGAACAAATCCAGATGGAGTTCTGAGGTCATTACTGGATCTATCAACAGGAGTCCAAGCGAGCTCGATATCAAA**TTACGCCCCGCCCTGCCACTCATCGCAGTACTGTTGTAATTCATTAAGCATTCTGCCGACATGGAAGCCATCACAAACGGCATGATGAACCTGAATCGCCAGCGGCATCAGCACCTTGTCGCCTTGCGTATAATATTTGCCCATGGTGAAAACGGGGGCGAAGAAGTTGTCCATATTGGCCACGTTTAAATCAAAACTGGTGAAACTCACCCAGGGATTGGCTGAGACGAAAAACATATTCTCAATAAACCCTTTAGGGAAATAGGCCAGGTTTTCACCGTAACACGCCACATCTTGCGAATATATGTGTAGAAACTGCCGGAAATCGTCGTGGTATTCACTCCAGAGCGATGAAAACGTTTCAGTTTGCTCATGGAAAACGGTGTAACAAGGGTGAACACTATCCCATATCACCAGCTCACCGTCTTTCATTGCCATACGAAATTCCGGATGAGCATTCATCAGGCGGGCAAGAATGTGAATAAAGGCCGGATAAAACTTGTGCTTATTTTTCTTTACGGTCTTTAAAAAGGCCGTAATATCCAGCTGAACGGTCTGGTTATAGGTACATTGAGCAACTGACTGAAATGCCTCAAAATGTTCTTTACGATGCCATTGGGATATATCAACGGTGGTATATCCAGTGATTTTTTTCTCCATTTTAGCTTCC**TTAGCTCCTGAAAATCTCGATAACTCAAAAAATACGCCCGGTAGTGATCTTATTTCATTATGGTGAAAGTTGGAACCTCTTACGTGCCCGATCAACTCGAGTGCCACCTGACGTCTAAGAAACCATTATTATCATGACATTAACCTATAAAAATAGGCGTATCACGAGGCAGAATTTCAGATAAAAAAAATCCTTAGCTTTCGCTAAGGATGATTTCTG

*pSB1C3-BCP* (3088)


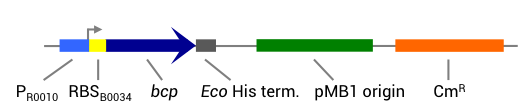


GAATTCGCGGCCGCTTCTAGAG**CAATACGCAAACCGCCTCTCCCCGCGCGTTGGCCGATTCATTAATGCAGCTGGCACGACAGGTTTCCCGACTGGAAAGCGGGCAGTGAGCGCAACGCAATTAATGTGAGTTAGCTCACTCATTAGGCACCCCAGGCTTTACACTTTATGCTTCCGGCTCGTATGTTGTGTGGAATTGTGAGCGGATAACAATTTCACACA**TACTAGAGAAAGAGGAGAAAATGAGTGTGATCGCTAAACAAATGACCTACAAGGTTTATATGTCAGGCACGGTCAATGGACACTACTTTGAGGTCGAAGGCGATGGAAAAGGTAAGCCCTACGAGGGGGAGCAGACGGTAAAGCTCACTGTCACCAAGGGCGGACCTCTGCCATTTGCTTGGGATATTTTATCACCACAGTGTCAGTACGGAAGCATACCATTCACCAAGTACCCTGAAGACATCCCTGACTATGTAAAGCAGTCATTCCCGGAGGGCTATACATGGGAGAGGATCATGAACTTTGAAGATGGTGCAGTGTGTACTGTCAGCAATGATTCCAGCATCCAAGGCAACTGTTTCATCTACCATGTCAAGTTCTCTGGTTTGAACTTTCCTCCCAATGGACCTGTCATGCAGAAGAAGACACAGGGCTGGGAACCCAACACTGAGCGTCTCTTTGCACGAGATGGAATGCTGCTAGGAAACAACTTTATGGCTCTGAAGTTAGAAGGAGGCGGTCACTATTTGTGTGAATTTAAAACTACTTACAAGGCAAAGAAGCCTGTGAAGATGCCAGGGTATCACTATGTTGACCGCAAACTGGATGTAACCAATCACAACAAGGATTACACTTCGGTTGAGCAGTGTGAAATTTCCATTGCACGCAAACCTGTGGTCGCCTAATAACCAGGCATCAAATAAAACGAAAGGCTCAGTCGAAAGACTGGGCCTTTCGTTTTATCTGTTGTTTGTCGGTGAACGCTCTCTACTAGAGTCACACTGGCTCACCTTCGGGTGGGCCTTTCTGCGTTTATATACTAGTAGCGGCCGCTGCAGTCCGGCAAAAAA**GGGCAAGGTGTCACCACCCTGCCCTTTTTCTTTAAAACCGAAAAGATTACTTCGCGTTAT**GCAGGCTTCCTCGCTCACTGACTCGCTGCGCTCGGTCGTTCGGCTGCGGCGAGCGGTATCAGCTCACTCAAAGGCGGTAATACGGTTATCCACAGAATCAGGGGATAACGCAGGAAAGAACATGTGAGCAAAAGGCCAGCAAAAGGCCAGGAACCGTAAAAAGGCCG**CGTTGCTGGCGTTTTTCCACAGGCTCCGCCCCCCTGACGAGCATCACAAAAATCGACGCTCAAGTCAGAGGTGGCGAAACCCGACAGGACTATAAAGATACCAGGCGTTTCCCCCTGGAAGCTCCCTCGTGCGCTCTCCTGTTCCGACCCTGCCGCTTACCGGATACCTGTCCGCCTTTCTCCCTTCGGGAAGCGTGGCGCTTTCTCATAGCTCACGCTGTAGGTATCTCAGTTCGGTGTAGGTCGTTCGCTCCAAGCTGGGCTGTGTGCACGAACCCCCCGTTCAGCCCGACCGCTGCGCCTTATCCGGTAACTATCGTCTTGAGTCCAACCCGGTAAGACACGACTTATCGCCACTGGCAGCAGCCACTGGTAACAGGATTAGCAGAGCGAGGTATGTAGGCGGTGCTACAGAGTTCTTGAAGTGGTGGCCTAACTACGGCTACACTAGAAGAACAGTATTTGGTATCTGCGCTCTGCTGAAGCCAGTTACCTTCGGAAAAAGAGTTGGTAGCTCTTGATCCGGCAAACAAACCACCGCTGGTAGCGGTGGTTTTTTTGTTTGCAAGCAGCAGATTACGCGCAGAAAAAAAGGATCTCAAGAAGATCCTTTGA**TCTTTTCTACGGGGTCTGACGCTCAGTGGAACGAAAACTCACGTTAAGGGATTTTGGTCATGAGATTATCAAAAAGGATCTTCACCTAGATCCTTTTAAATTAAAAATGAAGTTTTAAATCAATCTAAAGTATATATGAGTAAACTTGGTCTGACAGCTCGAGGCTTGGATTCTCACCAATAAAAAACGCCCGGCGGCAACCGAGCGTTCTGAACAAATCCAGATGGAGTTCTGAGGTCATTACTGGATCTATCAACAGGAGTCCAAGCGAGCTCGATATCAAA**TTACGCCCCGCCCTGCCACTCATCGCAGTACTGTTGTAATTCATTAAGCATTCTGCCGACATGGAAGCCATCACAAACGGCATGATGAACCTGAATCGCCAGCGGCATCAGCACCTTGTCGCCTTGCGTATAATATTTGCCCATGGTGAAAACGGGGGCGAAGAAGTTGTCCATATTGGCCACGTTTAAATCAAAACTGGTGAAACTCACCCAGGGATTGGCTGAGACGAAAAACATATTCTCAATAAACCCTTTAGGGAAATAGGCCAGGTTTTCACCGTAACACGCCACATCTTGCGAATATATGTGTAGAAACTGCCGGAAATCGTCGTGGTATTCACTCCAGAGCGATGAAAACGTTTCAGTTTGCTCATGGAAAACGGTGTAACAAGGGTGAACACTATCCCATATCACCAGCTCACCGTCTTTCATTGCCATACGAAATTCCGGATGAGCATTCATCAGGCGGGCAAGAATGTGAATAAAGGCCGGATAAAACTTGTGCTTATTTTTCTTTACGGTCTTTAAAAAGGCCGTAATATCCAGCTGAACGGTCTGGTTATAGGTACATTGAGCAACTGACTGAAATGCCTCAAAATGTTCTTTACGATGCCATTGGGATATATCAACGGTGGTATATCCAGTGATTTTTTTCTCCATTTTAGCTTCC**TTAGCTCCTGAAAATCTCGATAACTCAAAAAATACGCCCGGTAGTGATCTTATTTCATTATGGTGAAAGTTGGAACCTCTTACGTGCCCGATCAACTCGAGTGCCACCTGACGTCTAAGAAACCATTATTATCATGACATTAACCTATAAAAATAGGCGTATCACGAGGCAGAATTTCAGATAAAAAAAATCCTTAGCTTTCGCTAAGGATGATTTCTG

*pSB1K3-J04450* (3273 bp)


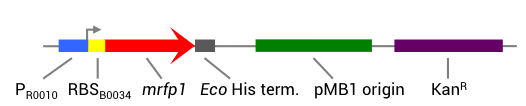


GAATTCGCGGCCGCTTCTAGAG**CAATACGCAAACCGCCTCTCCCCGCGCGTTGGCCGATTCATTAATGCAGCTGGCACGACAGGTTTCCCGACTGGAAAGCGGGCAGTGAGCGCAACGCAATTAATGTGAGTTAGCTCACTCATTAGGCACCCCAGGCTTTACACTTTATGCTTCCGGCTCGTATGTTGTGTGGAATTGTGAGCGGATAACAATTTCACACA**TACTAGAGAAAGAGGAGAAATACTAGATGGCTTCCTCCGAAGACGTTATCAAAGAGTTCATGCGTTTCAAAGTTCGTATGGAAGGTTCCGTTAACGGTCACGAGTTCGAAATCGAAGGTGAAGGTGAAGGTCGTCCGTACGAAGGTACCCAGACCGCTAAACTGAAAGTTACCAAAGGTGGTCCGCTGCCGTTCGCTTGGGACATCCTGTCCCCGCAGTTCCAGTACGGTTCCAAAGCTTACGTTAAACACCCGGCTGACATCCCGGACTACCTGAAACTGTCCTTCCCGGAAGGTTTCAAATGGGAACGTGTTATGAACTTCGAAGACGGTGGTGTTGTTACCGTTACCCAGGACTCCTCCCTGCAAGACGGTGAGTTCATCTACAAAGTTAAACTGCGTGGTACCAACTTCCCGTCCGACGGTCCGGTTATGCAGAAAAAAACCATGGGTTGGGAAGCTTCCACCGAACGTATGTACCCGGAAGACGGTGCTCTGAAAGGTGAAATCAAAATGCGTCTGAAACTGAAAGACGGTGGTCACTACGACGCTGAAGTTAAAACCACCTACATGGCTAAAAAACCGGTTCAGCTGCCGGGTGCTTACAAAACCGACATCAAACTGGACATCACCTCCCACAACGAAGACTACACCATCGTTGAACAGTACGAACGTGCTGAAGGTCGTCACTCCACCGGTGCTTAATAACGCTGATAGTGCTAGTGTAGATCGCTACTAGAGCCAGGCATCAAATAAAACGAAAGGCTCAGTCGAAAGACTGGGCCTTTCGTTTTATCTGTTGTTTGTCGGTGAACGCTCTCTACTAGAGTCACACTGGCTCACCTTCGGGTGGGCCTTTCTGCGTTTATATACTAGTAGCGGCCGCTGCAGTCCGGCAAAAAA**GGGCAAGGTGTCACCACCCTGCCCTTTTTCTTTAAAACCGAAAAGATTACTTCGCGTTAT**GCAGGCTTCCTCGCTCACTGACTCGCTGCGCTCGGTCGTTCGGCTGCGGCGAGCGGTATCAGCTCACTCAAAGGCGGTAATACGGTTATCCACAGAATCAGGGGATAACGCAGGAAAGAACATGTGAGCAAAAGGCCAGCAAAAGGCCAGGAACCGTAAAAAGGCCG**CGTTGCTGGCGTTTTTCCACAGGCTCCGCCCCCCTGACGAGCATCACAAAAATCGACGCTCAAGTCAGAGGTGGCGAAACCCGACAGGACTATAAAGATACCAGGCGTTTCCCCCTGGAAGCTCCCTCGTGCGCTCTCCTGTTCCGACCCTGCCGCTTACCGGATACCTGTCCGCCTTTCTCCCTTCGGGAAGCGTGGCGCTTTCTCATAGCTCACGCTGTAGGTATCTCAGTTCGGTGTAGGTCGTTCGCTCCAAGCTGGGCTGTGTGCACGAACCCCCCGTTCAGCCCGACCGCTGCGCCTTATCCGGTAACTATCGTCTTGAGTCCAACCCGGTAAGACACGACTTATCGCCACTGGCAGCAGCCACTGGTAACAGGATTAGCAGAGCGAGGTATGTAGGCGGTGCTACAGAGTTCTTGAAGTGGTGGCCTAACTACGGCTACACTAGAAGAACAGTATTTGGTATCTGCGCTCTGCTGAAGCCAGTTACCTTCGGAAAAAGAGTTGGTAGCTCTTGATCCGGCAAACAAACCACCGCTGGTAGCGGTGGTTTTTTTGTTTGCAAGCAGCAGATTACGCGCAGAAAAAAAGGATCTCAAGAAGATCCTTTGA**TCTTTTCTACGGGGTCTGACGCTCAGTGGAACGAAAACTCACGTTAAGGGATTTTGGTCATGAGATTATCAAAAAGGATCTTCACCTAGATCCTTTTAAATTAAAAATGAAGTTTTAAATCAATCTAAAGTATATATGAGTAAACTTGGTCTGACAGCTCGAGTCCCGTCAAGTCAGCGTAATGCTCTGCCAGTGTTACAACCAATTAACCAATTCTGATT**AGAAAAACTCATCGAGCATCAAATGAAACTGCAATTTATTCATATCAGGATTATCAATACCATATTTTTGAAAAAGCCGTTTCTGTAATGAAGGAGAAAACTCACCGAGGCAGTTCCATAGGATGGCAAGATCCTGGTATCGGTCTGCGATTCCGACTCGTCCAACATCAATACAACCTATTAATTTCCCCTCGTCAAAAATAAGGTTATCAAGTGAGAAATCACCATGAGTGACGACTGAATCCGGTGAGAATGGCAAAAGCTTATGCATTTCTTTCCAGACTTGTTCAACAGGCCAGCCATTACGCTCGTCATCAAAATCACTCGCATCAACCAAACCGTTATTCATTCGTGATTGCGCCTGAGCGAGACGAAATACGCGATCGCTGTTAAAAGGACAATTACAAACAGGAATCGAATGCAACCGGCGCAGGAACACTGCCAGCGCATCAACAATATTTTCACCTGAATCAGGATATTCTTCTAATACCTGGAATGCTGTTTTCCCGGGGATCGCAGTGGTGAGTAACCATGCATCATCAGGAGTACGGATAAAATGCTTGATGGTCGGAAGAGGCATAAATTCCGTCAGCCAGTTTAGTCTGACCATCTCATCTGTAACATCATTGGCAACGCTACCTTTGCCATGTTTCAGAAACAACTCTGGCGCATCGGGCTTCCCATACAATCGATAGATTGTCGCACCTGATTGCCCGACATTATCGCGAGCCCATTTATACCCATATAAATCAGCATCCATGTTGGAATTTAATCGCGGCCTGGAGCAAGACGTTTCCCGTTGAATATGGCTCATAA**CACCCCTTGTATTACTGTTTATGTAAGCAGACAGTTTTATTGTTCATGATGATATATTTTTATCTTGTGCAATGTAACATCAGAGATTTTGAGACACAACGTGGCTTTGTTGAATAAATCGAACTTTTGCTGAGTTGAAGGATCAGCTCGAGTGCCACCTGACGTCTAAGAAACCATTATTATCATGACATTAACCTATAAAAATAGGCGTATCACGAGGCAGAATTTCAGATAAAAAAAATCCTTAGCTTTCGCTAAGGATGATTTCTG

*pSB1K3-BCP* (3212 bp)


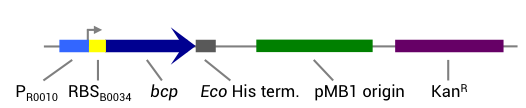


GAATTCGCGGCCGCTTCTAGAG**CAATACGCAAACCGCCTCTCCCCGCGCGTTGGCCGATTCATTAATGCAGCTGGCACGACAGGTTTCCCGACTGGAAAGCGGGCAGTGAGCGCAACGCAATTAATGTGAGTTAGCTCACTCATTAGGCACCCCAGGCTTTACACTTTATGCTTCCGGCTCGTATGTTGTGTGGAATTGTGAGCGGATAACAATTTCACACA**TACTAGAGAAAGAGGAGAAATACTAGATGAGTGTGATCGCTAAACAAATGACCTACAAGGTTTATATGTCAGGCACGGTCAATGGACACTACTTTGAGGTCGAAGGCGATGGAAAAGGTAAGCCCTACGAGGGGGAGCAGACGGTAAAGCTCACTGTCACCAAGGGCGGACCTCTGCCATTTGCTTGGGATATTTTATCACCACAGTGTCAGTACGGAAGCATACCATTCACCAAGTACCCTGAAGACATCCCTGACTATGTAAAGCAGTCATTCCCGGAGGGCTATACATGGGAGAGGATCATGAACTTTGAAGATGGTGCAGTGTGTACTGTCAGCAATGATTCCAGCATCCAAGGCAACTGTTTCATCTACCATGTCAAGTTCTCTGGTTTGAACTTTCCTCCCAATGGACCTGTCATGCAGAAGAAGACACAGGGCTGGGAACCCAACACTGAGCGTCTCTTTGCACGAGATGGAATGCTGCTAGGAAACAACTTTATGGCTCTGAAGTTAGAAGGAGGCGGTCACTATTTGTGTGAATTTAAAACTACTTACAAGGCAAAGAAGCCTGTGAAGATGCCAGGGTATCACTATGTTGACCGCAAACTGGATGTAACCAATCACAACAAGGATTACACTTCGGTTGAGCAGTGTGAAATTTCCATTGCACGCAAACCTGTGGTCGCCTAATAACCAGGCATCAAATAAAACGAAAGGCTCAGTCGAAAGACTGGGCCTTTCGTTTTATCTGTTGTTTGTCGGTGAACGCTCTCTACTAGAGTCACACTGGCTCACCTTCGGGTGGGCCTTTCTGCGTTTATATACTAGTAGCGGCCGCTGCAGTCCGGCAAAAAA**GGGCAAGGTGTCACCACCCTGCCCTTTTTCTTTAAAACCGAAAAGATTACTTCGCGTTAT**GCAGGCTTCCTCGCTCACTGACTCGCTGCGCTCGGTCGTTCGGCTGCGGCGAGCGGTATCAGCTCACTCAAAGGCGGTAATACGGTTATCCACAGAATCAGGGGATAACGCAGGAAAGAACATGTGAGCAAAAGGCCAGCAAAAGGCCAGGAACCGTAAAAAGGCCG**CGTTGCTGGCGTTTTTCCACAGGCTCCGCCCCCCTGACGAGCATCACAAAAATCGACGCTCAAGTCAGAGGTGGCGAAACCCGACAGGACTATAAAGATACCAGGCGTTTCCCCCTGGAAGCTCCCTCGTGCGCTCTCCTGTTCCGACCCTGCCGCTTACCGGATACCTGTCCGCCTTTCTCCCTTCGGGAAGCGTGGCGCTTTCTCATAGCTCACGCTGTAGGTATCTCAGTTCGGTGTAGGTCGTTCGCTCCAAGCTGGGCTGTGTGCACGAACCCCCCGTTCAGCCCGACCGCTGCGCCTTATCCGGTAACTATCGTCTTGAGTCCAACCCGGTAAGACACGACTTATCGCCACTGGCAGCAGCCACTGGTAACAGGATTAGCAGAGCGAGGTATGTAGGCGGTGCTACAGAGTTCTTGAAGTGGTGGCCTAACTACGGCTACACTAGAAGAACAGTATTTGGTATCTGCGCTCTGCTGAAGCCAGTTACCTTCGGAAAAAGAGTTGGTAGCTCTTGATCCGGCAAACAAACCACCGCTGGTAGCGGTGGTTTTTTTGTTTGCAAGCAGCAGATTACGCGCAGAAAAAAAGGATCTCAAGAAGATCCTTTGA**TCTTTTCTACGGGGTCTGACGCTCAGTGGAACGAAAACTCACGTTAAGGGATTTTGGTCATGAGATTATCAAAAAGGATCTTCACCTAGATCCTTTTAAATTAAAAATGAAGTTTTAAATCAATCTAAAGTATATATGAGTAAACTTGGTCTGACAGCTCGAGTCCCGTCAAGTCAGCGTAATGCTCTGCCAGTGTTACAACCAATTAACCAATTCTGATT**AGAAAAACTCATCGAGCATCAAATGAAACTGCAATTTATTCATATCAGGATTATCAATACCATATTTTTGAAAAAGCCGTTTCTGTAATGAAGGAGAAAACTCACCGAGGCAGTTCCATAGGATGGCAAGATCCTGGTATCGGTCTGCGATTCCGACTCGTCCAACATCAATACAACCTATTAATTTCCCCTCGTCAAAAATAAGGTTATCAAGTGAGAAATCACCATGAGTGACGACTGAATCCGGTGAGAATGGCAAAAGCTTATGCATTTCTTTCCAGACTTGTTCAACAGGCCAGCCATTACGCTCGTCATCAAAATCACTCGCATCAACCAAACCGTTATTCATTCGTGATTGCGCCTGAGCGAGACGAAATACGCGATCGCTGTTAAAAGGACAATTACAAACAGGAATCGAATGCAACCGGCGCAGGAACACTGCCAGCGCATCAACAATATTTTCACCTGAATCAGGATATTCTTCTAATACCTGGAATGCTGTTTTCCCGGGGATCGCAGTGGTGAGTAACCATGCATCATCAGGAGTACGGATAAAATGCTTGATGGTCGGAAGAGGCATAAATTCCGTCAGCCAGTTTAGTCTGACCATCTCATCTGTAACATCATTGGCAACGCTACCTTTGCCATGTTTCAGAAACAACTCTGGCGCATCGGGCTTCCCATACAATCGATAGATTGTCGCACCTGATTGCCCGACATTATCGCGAGCCCATTTATACCCATATAAATCAGCATCCATGTTGGAATTTAATCGCGGCCTGGAGCAAGACGTTTCCCGTTGAATATGGCTCATAA**CACCCCTTGTATTACTGTTTATGTAAGCAGACAGTTTTATTGTTCATGATGATATATTTTTATCTTGTGCAATGTAACATCAGAGATTTTGAGACACAACGTGGCTTTGTTGAATAAATCGAACTTTTGCTGAGTTGAAGGATCAGCTTCTAAGAAACCATTATTATCATGACATTAACCTATAAAAATAGGCGTATCACGAGGCAGAATTTCAGATAAAAAAAATCCTTAGCTTTCGCTAAGGATGATTTCTG
